# Amyloid Beta Is Internalized *via* Macropinocytosis, an HSPG- and Lipid Raft-Dependent and Rac1-Mediated Process

**DOI:** 10.3389/fnmol.2022.804702

**Published:** 2022-02-11

**Authors:** Keyoumu Nazere, Tetsuya Takahashi, Naoyuki Hara, Kazuki Muguruma, Masahiro Nakamori, Yu Yamazaki, Hiroyuki Morino, Hirofumi Maruyama

**Affiliations:** ^1^Department of Clinical Neuroscience and Therapeutics, Hiroshima University Graduate School of Biomedical and Health Sciences, Hiroshima, Japan; ^2^Department of Rehabilitation, Faculty of Rehabilitation, Hiroshima International University, Hiroshima, Japan; ^3^Department of Medical Genetics, Tokushima University Graduate School of Biomedical Sciences, Tokushima, Japan

**Keywords:** Alzheimer’s disease, amyloid – beta, HSPG, lipid raft, macropinocytosis, Rac1

## Abstract

Intracellular amyloid β peptide (Aβ) accumulation has drawn attention in relation to the pathophysiology of Alzheimer’s disease in addition to its extracellular deposition as senile plaque. Cellular uptake of extracellular Aβ is one of the possible mechanisms by which intracellular Aβ deposits form. Given the relevance of Aβ inside cells, it is important to understand the mechanism by which it is taken up by them. In this study, we elucidated that Neuro2A and SH-SY5Y cells internalize specifically oligomerized Aβ in a time- and dose-dependent manner. The depletion of plasma membrane cholesterol with methyl-β-cyclodextrin or treatment with trypsin diminished the internalization of oAβ, suggesting that the oAβ uptake might be both a lipid raft-dependent and heparan sulfate proteoglycan-mediated process. Treatment with a macropinocytosis inhibitor (ethylisopropyl amiloride and wortmannin) also drastically reduced the uptake of oligomer-Aβ (oAβ). oAβ-treated cells exhibited an increase in Rac1 activity, indicating that macropinocytosis induced by oAβ is regulated by these small GTPases. These findings suggest that macropinocytosis is a major endocytic route through which oAβ42 enters cells.

## Introduction

Alzheimer’s disease (AD) is the most common form of dementia, which is histopathologically characterized by the presence of senile plaques and neurofibrillary tangles (NFTs; Belyaev et al., 2010; Viola and Klein, 2015; Frost and Li, 2017). Senile plaques are extracellular deposits of aggregated amyloid β peptide (Aβ), while NFTs are intracellular aggregates composed mainly of hyperphosphorylated tau protein (Serrano-Pozo et al., 2011). Aβ is a 40- or 42-amino-acid peptide produced through the cleavage of amyloid precursor protein by β- and γ-secretase (Belyaev et al., 2010; Serrano-Pozo et al., 2011; Frost and Li, 2017). In addition to both ends of monomer state and fibril, distinct intermediate species of Aβ42 in the form of low-molecular-weight oligomers, such as dimers, trimers, and larger spherical oligomers, so-called Aβ-derived diffusible ligands, have been reported (Serrano-Pozo et al., 2011). The overproduction of Aβ42 or impairment of its proper clearance is pivotal in the appearance of senile plaques, which is thought to be an event leading to the formation of NFTs (Singh et al., 2016). Apart from senile plaques as extracellular Aβ42 deposits, increasing evidence has demonstrated the pathological relevance of intracellular Aβ42 (Takahashi et al., 2004; Chafekar et al., 2008; Ansari et al., 2009; Bharadwaj et al., 2009; Thal et al., 2015); however, the precise mechanism that links NFT formation and intracellular as well as extracellular Aβ42 remains unclear. Regarding the mechanism behind the appearance of intracellular Aβ42, several studies have reported that the generation of Aβ42 might take place inside intracellular vesicles such as endosomes (Zhang and Song, 2013; Schützmann et al., 2021). Another plausible manner by which intracellular Aβ42 will appear is that cells actively take up the secreted Aβ42 and traffic it to multivesicular bodies (Friedrich et al., 2010). Although Aβ42 might appear inside cells not in an exclusive manner, information on the mechanism by which Aβ42 can be internalized should be valuable in the search for future therapeutics for AD.

Living cells can endocytose a diverse array of extracellular materials such as solute molecules, nutrients, and antigens, and the vast majority of viruses hijack these mechanisms at the initial step of infection (Doherty and McMahon, 2009). Among a variety of types of endocytosis, clathrin-dependent endocytosis is the most extensively studied, using transferrin as a representative marker (Mayor and Pagano, 2007). Recently, findings on macropinocytosis among the rest of endocytosis (non-clathrin endocytosis) have emerged. Macropinosome formation is driven by the rearrangement of actin filaments underlying the plasma membrane, which is regulated by PI3K, Rac1, and Arf6 (Swanson and Watts, 1995). Human immunodeficiency virus (HIV) virion and the technique of gene delivery of lipoplexes utilize macropinocytosis (Letoha et al., 2013). Interestingly, the oligomer of human amylin, a 37-amino-acid peptide produced by pancreatic beta cells, plays a role in the pathogenesis of type 2 diabetes mellitus, as Aβ does in AD. In the case of amylin, oligomeric amylin has been shown to be taken up into islet cells *via* macropinocytosis, which raises the possibility that the mechanism of oligomer-Aβ (oAβ)42 internalization is the same as that for amylin (Clark and Nilsson, 2004; Trikha and Jeremic, 2011, 2013).

To test this hypothesis, we examined the dynamics of Aβ42 internalization using culture cells, including non-neuronal cells, and found that cells take up Aβ42 in a time- and concentration-dependent manner. We next found that the uptake process requires both intact heparan sulfate proteoglycan (HSPG)s and lipid rafts. We also discovered that the macropinocytosis process regulated by Rac1 is involved in the uptake of oligomeric Aβ42 (oAβ42).

## Methods and Reagents

### Reagents

FITC-labeled Aβ42 was purchased from GL Biochem Ltd. (Shanghai, China); TMR-labeled Aβ42, FITC-dextran (MW: 70,000), a marker for the macropinocytosis pathway, FITC-Cholera Toxin B subunit (CTB), a marker of Lipid raft, heparin sodium salt an inhibitor of HSPGs, and methyl-β-cyclodextrin (MβCD) that can disrupt lipid raft, were obtained from Sigma-Aldrich (St. Louis, MO, USA); ethylisopropyl amiloride (EIPA) a blockef of macropinocytosis, SecinH3, an indirect inhibitor of Arf6, Grassofermata (NAV 2729) a direct inhibitor of Arf6, and EHT1864, a Rac1 inhibitor of were purchased from Cayman chemical (Ann Arbor, MI, USA); dynasore (ab120192), a dynamin inhibitor was from Abcam (Cambridge, MA, USA), Alexa Fluor 488 transferrin and phalloidin were obtained from Invitrogen (Carlsbad, CA, USA); wortmannin, a phosphoinositide 3-kinase inhibitor, Dulbecco’s Modified Eagle Medium (DMEM) cell culture medium, phenol red-free Ham’s F12 Medium, 0.25% trypsin-EDTA, and 1,1,1,3,3,3-hexafluoro-2-propanol (HFIP) were from Fujifilm Wako Pure Chemical Corporation (Osaka, Japan); fetal bovine serum was from COSMO BIO (Tokyo, Japan); and Hoechst 33342 and 4’,6-diamidino-2-phenylindole (DAPI), were from Dojindo (Kumamoto, Japan). G-LISA (ELISA-based GTPase activation assay) kits obtained from Cytoskeleton (Denver, CO, USA) were used to measure the activities of Rac1 and Arf6.

### Preparation of Amyloid Beta Monomers and Oligomers

We first allowed lyophilized Aβ42 to equilibrate at room temperature for 30 min, followed by the addition of 200 μl of HFIP to obtain a 1 mM solution and vortexing the solution for a few seconds. After evaporation of HFIP overnight in a fume hood, Aβ42 peptide in microtubes was transferred to a vacuum concentrator and dried down for 1 h without heating to remove any remaining traces of HFIP and moisture. The dried peptide films in microtubes were stored with a desiccant at -20°C. The stored peptide films were allowed to come to room temperature at the time of usage. Subsequently, the peptide film was reconstituted in dimethylsulfoxide (DMSO) and the solution was sonicated for 10 min in a bath sonicator to make 5 mM Aβ42 solution. Ice-cold H_2_O was added to obtain a monomeric Aβ42 (mAβ42) solution with a final concentration of 100 μM, vortexed for 15 s, and used immediately. As for the preparation of oAβ42 (100 μM), dried peptide film of Aβ42 was dissolved in DMSO, mixed well with the phenol red-free Ham’s F12 medium, and incubated for 6 h at 4°C.

The 5 μM of TMR-Aβ42 samples prepared as monomer or oligomer were subjected to non-reducing SDS-PAGE using 16.5% Tris-Tricine gel. After electrophoresis, the fluorescence in the gel was observed on a UV illuminator to check the monomeric or oligomeric status.

### Cell Cultures and Treatments

Neuro2A, SH-SY5Y, HeLa, and HEK293T cells were cultured in DMEM supplemented with 10% (v/v) fetal bovine serum and 1% penicillin/streptomycin at 37°C in a humidified incubator with 5% CO_2_. Cells were passaged bi-weekly. Passages 2–10 were used for all of the experiments. Cells were plated at a density of 50,000 cells/well and cultured for 24 h before the treatment with oAβ42 unless otherwise indicated. In the experiments using inhibitors, cells were pretreated with EIPA (80 μM), wortmannin (300 nM), or dynasore (80 mM) for 1 h, or 10 mM or 20 mM MβCD, and 50 ng/ml heparin sodium salt for 30 min, followed by the addition of oAβ42 at the indicated concentrations for 90 min.

To test whether Arf6, a small GTPase, is involved in the cellular uptake of oAβ42, Neuro2A cells were cultured with oAβ42 in the presence of the direct or indirect inhibitor of Arf6 (NAV 2729 at 5 μM or SecinH3 at 150 μM for 1 h), followed by washing with chilled PBS, fixing, and then staining of nuclei with DAPI. Neuro2A cells and SH-SY5Y cells were first incubated with 5 μM EHT1864, a Rac1 inhibitor, at 37°C for 60 min, and then treated with oAβ42 for 90 min.

To explore the effect of the mode of culture passage on the entry of oAβ42, two types of culture dishes were prepared. In one type, cells were passaged using a scraper to dissociate them and maintain the cell surface HSPGs. In the other dishes, cells were dissociated by 8 min of treatment with 0.25% trypsin-EDTA to cleave HSPGs. Each dissociated floating cell was treated with oAβ42 for 90 min, centrifuged at 500× *g* for 3 min, and then washed twice with DMEM to discard the free oAβ42. The cells were further incubated for 24 h before microscopic examination.

To visualize actin filaments with Alexa-488 phalloidin, cells were treated with oAβ42 for 90 min, followed by washing with chilled PBS, fixing with 4% paraformaldehyde (PFA) for 15 min, and then processing with permeabilization and blocking, using a mixed solution of 0.5% saponin and 5% normal goat serum, 137 mM sodium glutamate, 2 mM MgCl_2_, and 1 mg/ml BSA, pH 6.8, for 4 h at 4°C. After two final washes in ice-cold PBS, coverslips were mounted on slides using DAPI.

### Confocal Microscopy

The cells were incubated with TMR- or FITC-labeled oAβ42 and the FITC-labeled endocytosis marker dextran at 37°C for 90 min. After washing, the cells were fixed with 4% PFA and permeabilized. Images were obtained using a confocal laser scanning microscope (FV1000-D IX81; Olympus, Tokyo, Japan). Three 50 mW solid-state lasers (405, 488, and 568 nm) coupled to an acoustic-optical tunable filter were used. The degree of internalization of oAβ42 and its accumulation in Neuro2A and SH-SY5Y cells were determined using ImageJ. The integrated amount of oAβ42 accumulated in the cell corrected by the background represented the total ligand uptake. Briefly, the cell contour in each plane was determined by increasing the brightness of the image. In some cases, brightfield images were also taken to help identify cell contours. Upon various pharmacological treatments, all intact cells in each image area were evaluated for quantitative oAβ42 uptake, based on the FITC signal intensity of the green channel and the TMR signal of the red channel. Briefly, regions of interest corresponding to oAβ42 and dextran deposits that stained positively for DAPI were created by thresholding (auto-thresholding). Among several methods for auto-thresholding in ImageJ, we used default and B&W switches to minimize background noise and convert the original grayscale image to a binary image.

### Measurements of Small GTPase Activities

Several GTPases have been shown to be activated in the course of macropinocytosis. To explore the possibility of this occurring in the process of oAβ42 internalization, we assessed the changes in the activity of small GTPases (Rac1 and Arf6) under oAβ42 treatment. Neruo2A cells were incubated with oAβ42 in 35 mm culture dishes for 7, 15, and 30 min (for the Arf6 activation assay, we additionally prepared 3 and 5 min culture dishes) as distinctive groups, and the cell lysates were collected. Rac1 and Arf6 activities in the lysates were measured using the respective G-LISA absorbance-based activation assay kits (Cytoskeleton Inc., Denver, CO, USA). Briefly, Neuro2A cells were lysed in a buffer provided by the manufacturer, and clarified lysates were incubated in Rac1/Arf6-GTP-binding protein-coated wells. Following washes to remove nonspecific binding, bound Rac1/Arf6-GTP was detected using anti-Rac1 or Arf6 primary and HRP-linked secondary antibodies. The read-out was obtained at a wavelength of 490 nm.

### Statistics

Data are presented as mean ± standard error of the mean (SEM) of three replicate experiments. Statistical analysis was performed by one-way analysis of variance (ANOVA) using JMP Pro15 (SAS Institute, Cary, NC, USA), followed by multiple comparisons with Dunnett’s test to test for differences in mean fluorescent intensity between samples and differences in the activities of GTPases (Rac1 and Arf6) among groups.

## Results

### Time and Dose Dependence of Cellular Uptake of Amyloid Beta

**Figure 1:**
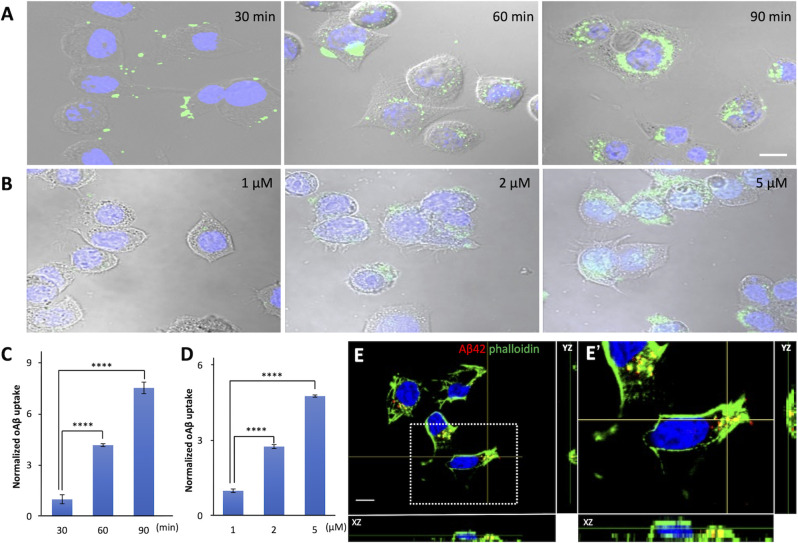
The time and dose dependence of cellular uptake of amyloid beta in neuroblastoma cells. **(A)** Neuro2A cells were allowed to internalize 5 μM FITC-labeled oAβ42 for 30, 60, and 90 min, followed by washing and fixation. Nuclei were stained with DAPI (blue). **(B)** Neuro2A cells were treated with 1, 2, and 5 μM oAβ42 for 90 min and nuclei were stained with DAPI. All experiments were repeated three times. Quantification of the fluorescence intensity shows **(C)** a time-dependent increase in intracellular oAβ42 and **(D)** a dose-dependent increase in intracellular oAβ42. All experiments were repeated three times. Data are presented as mean ± SEM, and data are presented as mean fluorescence normalized to 30 min or 1 μM. Error bars indicate standard error (*n* = 3). Significance was established using ANOVA followed by the Dunnett–Square test. Scale bar = 10 μm. **(E)** The two orthogonal views (XZ and YZ) on confocal microscopy showed that oAβ42 labeled with TMR was surrounded by actin (arrows) labeled with phalloidin (green). The nuclei were visualized with DAPI (blue). An enlarged view of the boxed region is shown in **(E’)**. Scale bar = 10 μm. ****p < 0.0001.

Intracellular Aβ42 has attracted substantial attention over the past few years (Chafekar et al., 2008; Dorostkar et al., 2015). One of the possible reasons why Aβ42 is observed in cells is that extracellularly generated Aβ42 enters them. To test this possibility, we examined the dynamics of internalization of oAβ42 peptides of different concentrations using Neuro2A cells. The culture medium with 5 μM FITC-labeled oAβ42 was replaced with fresh DMEM without peptide at the time points of 30, 60, and 90 min. The representative photographs in Figure 1 illustrate an increase in intracellularly accumulated punctate oAβ42. In the time-dependent uptake experiment (Figures 1A–C), the level of internalized oAβ42 was 4.2-fold higher at 60 min [95% confidence interval (CI) 3.99–4.36; *p* < 0.0001] and 7.5-fold higher at 90 min (95% CI 7.19–7.87; *p* < 0.0001) than that at 30 min (31.39 ± 8.3).

Next, we treated the cells with different concentrations of FITC-labeled oAβ42 (1, 2, and 5 μM) for 90 min at 37°C. In this dose-dependent uptake experiment (Figures 1B–D), compared with 1 μM (50.47 ± 3.6), the signal intensity of oAβ42 at 2 μM increased by 2.8- fold (95% CI 2.65–2.82; *p* < 0.0001) and that at 5 μM by 4.8-fold (95% CI 4.60–4.88; *p* < 0.0001). Taking these findings together, oAβ42 is taken up into cells in a time- and dose-dependent manner. We next tested the ability of 5 μM mAβ42 to enter Neuro2a cells after 90 min of incubation; however, mAβ42 exhibited a capacity for internalization only comparable to a lower concentration (1 μM) of oAβ42 (Supplementary Figures 1A,B). Next, both mAβ42 and oAβ42 were placed in a Tris-Tricine gel to check whether they were in a monomeric or oligomeric form. Here, peptides were dissolved in gel loading buffer and electrophoresed in a 16.5% Tris-Tricine gel at 100 V for 120 min, followed by observation under 312 nm UV light (Supplementary Figure 1C).

To confirm that oAβ was taken up into cells, we analyzed confocal micrographs three-dimensionally. The two orthogonal views from different planes (XZ and YZ) of the images showed that oAβ42 labeled with TMR localized not on the surface of cell membranes but inside the cells. Notably, the oAβ42 inside the cells was surrounded by actin, as visualized by phalloidin (Figures 1E,E’).

### Amyloid Beta Oligomer Uptake Is Dependent on Intact Lipid Rafts

**Figure 2:**
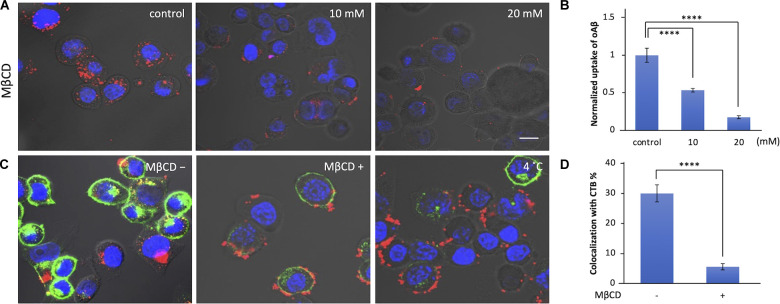
Effect of lipid raft disruption with cyclodextrin on oAβ42 uptake. **(A)** Neuro2A cells were pretreated with MβCD (10 or 20 mM) for 30 min at 37°C; then, the cell culture medium was exchanged with that with 5 μM oAβ42 and incubated for 90 min at 37°C. Nuclei were visualized with Hoechst 33342 (blue). **(B)** Quantification of the fluorescence intensity shows the uptake of oAβ42, and data are presented as mean fluorescence normalized to control. Significance was established using ANOVA followed by the Dunnett–Square test. Data are presented as mean ± SEM. All experiments were repeated three times. **(C)** Neuro2A cells were incubated with or without 10 mM MβCD for 30 min at 37°C, followed by incubation with 20 μg/ml CTB for 10 min at 4°C. Subsequently, cells were further incubated at 37°C for 90 min in combination with oAβ42 (5 μM). Alternatively, Neuro2A cells were pretreated with 5 μM oAβ42 and 20 μg/ml CTB at 4°C for 10 min, and then at 37°C for 90 min. **(D)** Quantification of the colocalization of oAβ42 and CTB (colocalization/total) in the absence or presence of MβCD (10 mM). All experiments were repeated three times. Data are presented as mean ± SEM. Significance was established by Student’s t-test. Scale bars = 10 μm. ****p < 0.0001.

Lipid rafts are highly organized membrane domains enriched with cholesterol/sphingolipid and play roles in various types of endocytosis in the initial binding steps (Nichols, 2003; Rushworth and Hooper, 2010; Bieberich, 2018). We examined whether lipid rafts are also involved in the cellular uptake of oAβ42 using MβCD, which depletes cholesterol from the membrane, thereby disrupting lipid rafts. As shown in Figures 2A,B, MβCD decreased the uptake of oAβ42 (46.6% with 10 mM and 82.5% with 20 mM, respectively; *p* < 0.0001) in a manner dependent on the concentration of MβCD. This indicates that the lipid rafts are critical in the process of oAβ42 internalization.

To visualize lipid rafts, Neuro2A cells were incubated in the presence of CTB, a nontoxic portion of cholera toxin with high affinity to the GM1 ganglioside (Gupta and DeFranco, 2003). As shown in Figures 2C,D, we observed that 30.0% of CTB colocalized with oAβ42; in addition, in the presence of MβCD, the colocalization rate was significantly reduced to 5.6%. Alternatively, Neuro2A cells were simultaneously treated with oAβ42 and CTB at 4°C for 10 min to facilitate the staining of lipid rafts, and then at 37°C for 90 min (Figure 2C, right). In this case, prolonged attachment of oAβ42 to the surface of cells would facilitate the further accumulation of oAβ42 into aggregates, which appeared as deposits. Lowering the temperature might block molecules that are involved in the internalization of oAβ42.

### HSPGs Mediate Amyloid Beta Oligomer Uptake

**Figure 3:**
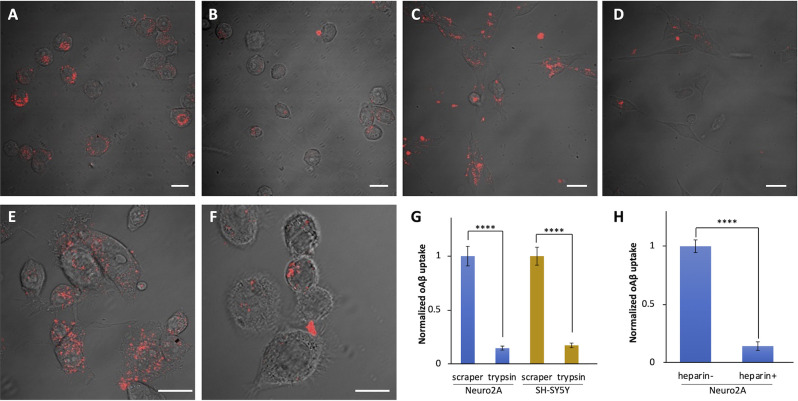
Impaired uptake of oAβ42 by the treatment with heparin and trypsin. **(A,B)** Neuro2A cells were dissociated using a scraper **(A)** or by 8 min of treatment with 0.25% trypsin-EDTA **(B)**. After treatment with oAβ42 in 1.5 ml microcentrifuge tubes for 90 min, cells were washed and incubated for 24 h at 37°C in a medium free of oAβ42. The next day, cells were fixed and observed under a confocal microscope. SH-SY5Y cells were treated in the same manner using a scraper **(C)** or trypsin **(D)**. Neuro2A cells were cultured in the absence **(E)** or presence **(F)** of heparin (50 ng/ml) for 30 min; then, oAβ42 (5 μM) was added for 90 min and observed under a confocal microscope. Scale bars = 10 μm. **(G,H)** Fluorescent intensity of three independent experiments shows a marked reduction in the uptake of oAβ42 in the presence of trypsin and heparin. Data are presented as mean fluorescence normalized to scraper or heparin. Data are presented as mean ± SEM. Significance was established by Student’s t-test. ****p < 0.0001.

Heparan sulfate proteoglycans (HSPGs) on the cell surface can function as co-receptors of the diverse array of receptors and actively internalize various substrates into the cells. We examined whether oAβ42 internalization is an HSPG-mediated process using trypsin and heparin, given that trypsin can cleave HSPGs and heparin can block HSPGs. In the experiment using trypsin, we dissociated cells with 0.25% trypsin-EDTA at culture passage and treated them with oAβ42. Subsequently, the cells were collected and washed twice with medium to discard unincorporated oAβ42, followed by further incubationof the cells attached to the bottom of the dishes. For comparison, another group of cells was dissociated using a scraper to keep HSPGs intact during culture passage. As shown in Figure 3G, quantitative analysis of the results showed a clear reduction in the amount of oAβ42 inside the cells passaged using trypsin in both Neuro2A cells (scraper: 224.0 ± 20.3 vs. trypsin: 33.4 ± 4.1, *p* < 0.0001; Figures 3A,B) and SH-SY5Y cells (scraper: 214.8 ± 17.8 vs. trypsin: 37.4 ± 4.5, *p* < 0.0001; Figures 3C,D). Next, we used heparin as a blocker for HSPGs to examine whether it can interfere with the oAβ42 uptake activity of Neuro2A cells (Figures 3E,F). We found that 30 min of pretreatment with heparin significantly decreased intracellular oAβ42 by 6.9-fold compared with that of untreated cells (treated: 34.9 ± 8.8 vs. untreated: 241.6 ± 13.1, *p* < 0.0001; Figure 3H).

### Amyloid Beta Oligomer Is Internalized Into Cells by Macropinocytosis

**Figure 4:**
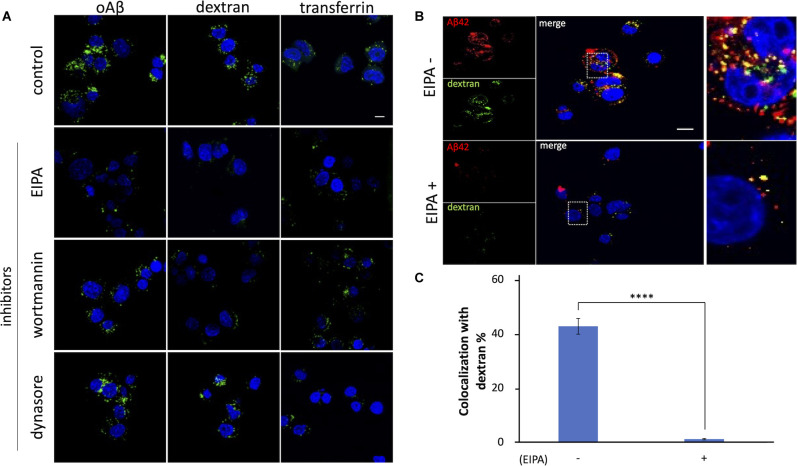
Effects ofinhibitors on the internalization of oAβ42 in Neuro2A cells.**(A)** Neuro2A cells were mock-treated or pretreatedwith 80 μM EIPA, 300 nM wortmannin, or 80 mM dynasore for 1 h,followed by incubation with 5 μM oAβ42, 2.5 mg/mldextran, or 50 μg/ml transferrin for an additional 90 min at37°C, respectively. Nuclei were visualized with DAPI (blue).Scale bar = 10 μm. **(B)** Neuro2A cells were treated with 5 μM TMR-oAβ42 (red) and 2.5 mg/ml FITC-dextran (green) for 90 min in the absence or presence of EIPA. Yellow indicates colocalization. Nuclei were visualized with DAPI (blue). Enlarged views of the boxed region are shown in **(B)** (far right line). Scale bar = 10 μm. **(C)** Quantification of the rate of coexistence of oAβ42 and dextran (colocalization/total) shows a marked reduction in the presence of EIPA. Experiments were repeated three times. Data are presented as mean ± SEM. Significance was established by Student’s t-test. ****p < 0.0001.

Evidence has demonstrated that, in neuronal cells, macropinocytosis is mediated by HSPGs (Holmes et al., 2013). It has also been reported that both HSPGs and lipid rafts are involved in the internalization of proteins released by eosinophils (Fan et al., 2007) and the HIV Tat protein transduction domain (Imamura et al., 2011). Since our results showed that oAβ42 is internalized in a lipid raft-dependent and HSPG-mediated manner, we examined whether oAβ42 was taken up by cells *via* macropinocytosis. Neuro2A cells were pretreated for 1 h with EIPA, a Na^+^/H^+^ exchanger inhibitor, or wortmannin, a phosphoinositide 3-kinase inhibitor, both of which can block macropinocytosis. Subsequently, cells were cultured in a medium containing oAβ42 with dextran or transferrin. The pretreatment with EIPA or wortmannin attenuated the signals from oAβ42 and dextran (Figure 4A). In contrast, these pretreatments did not noticeably change the amount of intracellular transferrin. To test the possible role of clathrin-dependent endocytosis in the internalization of oAβ42, cells were treated with dynasore, a cell-permeable inhibitor of dynamin. As a result, dynasore decreased the uptake of transferrin, but not that of oAβ42 and dextran (120 cells observed for each ligand). The same experiment using SH-SY5Y cells showed similar results (Supplementary Figure 2A). The rate of coexistence of oAβ42 and dextran (colocalization/total) after 90 min of treatment was 42.9% (Figures 4B,C) in the absence of EIPA, but it decreased significantly to 2.9% in the presence of EIPA (*p* < 0.0001). These results suggest that oAβ42 was internalized in Neuro2A cells *via* macropinocytosis. Next, we confirmed the internalization of oAβ42 by other cells to generalize our findings beyond the neuronal cells. We treated SH-SY5Y, HeLa, HEK293T, as well as Neuro2A cells with fluorescently labeled oAβ42. In all cells tested, oAβ42 was observed as punctate spots throughout the cell bodies (Supplementary Figures 2B,C).

### Small GTPase Rac1 Is Involved in Amyloid Beta Oligomer Internalization

**Figure 5:**
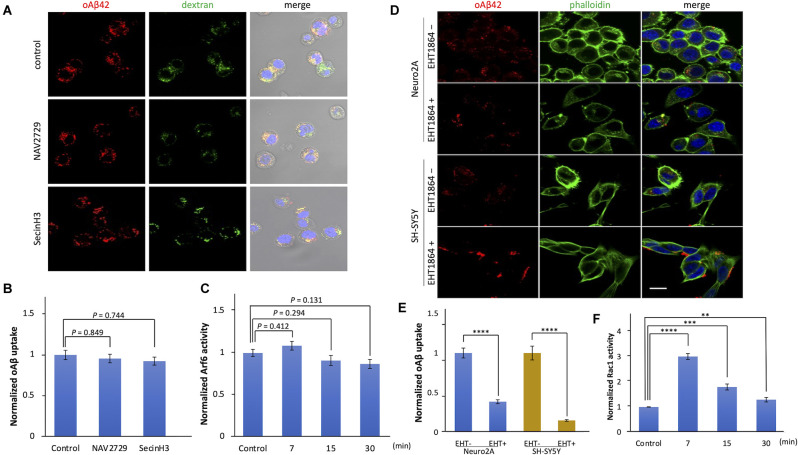
The effects of Arf6 and Rac1 inhibitors on the uptake of oligomeric amyloid beta. **(A)** Cells were treated with NAV 2729 and SecinH3 for 1 h, followed by incubation with 5 μM TMR-oAβ42 (red) and 2.5 mg/ml FITC-dextran (green) for 90 min at 37°C. Nuclei were visualized with DAPI (blue). Scale bar = 10 μm. **(B)** Quantification of the uptake of oAβ42 by confocal microscopy in the presence of the Arf6 inhibitors, and data are presented as mean fluorescence normalized to control (*n* = 3). Significance was established by ANOVA followed by the Dunnett–Square test. **(C)** The change in Arf6 activity in Neuro2A cells by the treatment of oAβ42 was measured using G-LISA. Normalized data are presented as mean ± SEM. Significance was established by ANOVA followed by the Dunnett–Square test. **(D)** Actin and nuclei were visualized with Alexa Fluor 488 phalloidin conjugates (green) and DAPI, respectively. Scale bar = 10 μm. **(E)** Quantification of the uptake of oAβ42 by confocal microscopy in the presence of EHT1864. Data are presented as mean fluorescence normalized to without EHT (*n* = 3). Significance was established by Student’s t-test. **(F)** The change in Rac-1 activity in Neuro2A cells upon oAβ42 treatment was measured using G-LISA. Normalized data are presented as mean ± SEM. Significance was established by ANOVA followed by the Dunnett–Square test. **p < 0.01; ***p < 0.001; ****p < 0.0001.

Macropinocytosis is regulated by small GTPases such as ADP ribosylation factor protein 6 (Arf6) and Rac1 (Désiré et al., 2005; Tang et al., 2015). To examine whether these GTPases are involved in the internalization of oAβ42, we used specific inhibitors. First, we treated Neuro2A cells with Arf6 inhibitors, NAV 2729, or an indirect inhibitor, SecinH3, and then incubated them with oAβ42. SecinH3 is a cytohesin-specific small-molecule inhibitor that blocks the activation of Arf6 through the inhibition of ARNO. In the presence of these inhibitors, the amount of intracellular oAβ42 did not significantly decrease (NAV 2729, *p* = 0.849; SecinH3, *p* = 0.744; Figures 5A,B), suggesting that Arf6 does not take part in oAβ42 uptake. In contrast, EHT1864, an inhibitor of Rac1, decreased intracellular oAβ42 in Neuro2A and SH-SY5Y cells by 61.4% and 85.1% respectively (Neuro2A, *p* < 0.0001; SH-SY5Y, *p* < 0.0001; Figures 5D,E). Taken together, these studies indicate that the internalization of oAβ42 is dependent solely on Rac1 activity. Next, we studied the change in activities of Arf6 and Rac1 during the entry of oAβ42. Lysates from Neuro2A cells incubated with 5 μM oAβ42 for 7, 15, and 30 min were subjected to Rac1 G-LISA assay. We used a lysate concentration of 0.5 mg/ml for the Rac1 G-LISA to keep the assay in the linear range. The signal was read by measuring absorbance at 490 nm using a microplate spectrophotometer. Wells with lysis buffer alone were designated as blanks for the assay. The results indicated that Rac1 activity at the time of 7 min showed a maximum increase of 3.0-fold (95% CI 2.89–3.12, *p* < 0.0001) compared with the control (1.13 ± 0.12), and then gradually decreased with increasing incubation (15 min, 1.79 ± 0.12, *p* < 0.001; 30 min, 1.29 ± 0.09, *p* = 0.008; Figure 5F). However, Arf6 activity upon treatment with oAβ42 at the times of 7 (0.14 ± 0.006, *p* = 0.412), 15 (0.13 ± 0.004, *p* = 0.294), and 30 min (0.12 ± 0.004, *p* = 0.131) did not change compared with that of the control (0.13 ± 0.006; Figure 5C). The activation and inhibition assays suggested that Arf6 is not involved in the endocytosis of oAβ42.

## Discussion

To better understand the pathological association of extracellular Aβ42 deposition and neurodegeneration, we investigated the mode of Aβ42 entry into culture cells using synthetic Aβ42, based on the assumption that intracellular Aβ42 plays a role in neurodegeneration. Our study showed that oAβ42 is distinctively internalized *via* Rac1-mediated macropinocytosis, which requires HSPGs and lipid rafts. Although the pathological significance of this is not yet fully understood, there is a growing body of evidence that Aβ42 exists not only extracellularly but also intracellularly (Hu et al., 2009; Bayer and Wirths, 2010; Lai and McLaurin, 2010; Omtri et al., 2012; Ji et al., 2016; Wesén et al., 2017; Welikovitch et al., 2018). With regard to intracellular Aβ42, there is a possibility that Aβ42 is formed inside the cell and is secreted, resulting in senile plaque formation. Another source of senile plaque might be the dead cells bearing intracellular oAβ. In accordance with this notion, Tang et al. (2015) demonstrated that amyloid precursor protein is directly delivered *via* macropinocytosis to the lysosome where Aβ42 is generated from amyloid precursor protein (Tam et al., 2016). Another possibility is that the endocytic process internalizes extracellularly formed Aβ42. The presence of Aβ42 in the multivesicular bodies (Takahashi et al., 2002) supports both above-mentioned notions since multivesicular bodies are a central node that connects both the endosome–lysosome pathway and the exosome secretion pathway. A recent study demonstrated that amyloid precursor protein functions as adhesion molecules bridging presynaptic and postsynaptic membranes. Enhanced synaptic activity leads to increased APP processing and Aβ generation (Stahl et al., 2014). Our results demonstrate that the uptake of extracellular oAβ42 is one probable explanation for the presence of intracellular oAβ42.

Endocytosis encompassing a variety of routes is critical for living cells to take up extracellular materials (El-Sayed and Harashima, 2013). Clathrin-dependent endocytosis is the type of endocytosis that has been most extensively studied to date. Transferrin is well known to be incorporated *via* clathrin-dependent endocytosis driven by the motor protein dynamin (Mayor and Pagano, 2007). Dynasore, a dynamin inhibitor, was found to inhibit the endocytosis of transferrin but not oAβ42, indicating that clathrin-dependent endocytosis plays only a minor role. In our study, internalized oAβ42 was surrounded by actin filaments visualized by phalloidin. The pretreatment of Rac1 inhibitors significantly reduced the internalization of both oAβ42 and dextran, whereas the G-LISA assay showed that Rac1 was activated by oAβ42. Regarding Rac1 activity, Borin et al. (2018) reported no change in the activity at the time points of 1, 3, 6, and 24 h after oAβ42 treatment. However, their results are not incompatible with our findings since we observed that the level of Rac1 activity returned to the basal level as early as 30 min. Rather, our results are in line with the fact that Rac1 is involved in the early stage of macropinocytosis (Kunita et al., 2007).

Eosinophil cationic protein (ECP), a superfamily member related to human RNase and asthma pathology, was reported to be another molecule incorporated *via* macropinocytosis. It was also shown that HSPGs and lipid rafts are indispensable for the internalization of ECP (Fan et al., 2007). Specifically, cell surface-bound HSPGs (syndecan, glypican) act as coreceptors for various growth factor receptors and several viruses (Sarrazin et al., 2011; Cagno et al., 2019). Interestingly, syndecan is found in senile plaques and neurofibrillary tangles of AD-affected brains (Verbeek et al., 1999). These findings prompted us to examine the dependence of oAβ42 on HSPGs and lipid rafts. The pretreatment of trypsin or coincubation with heparin to interfere with HSPGs reduced the amount of intracellular oAβ42. Likewise, cellular uptake of oAβ42 was decreased in the presence of MβCD that destroys lipid rafts. The dependence on lipid rafts was previously reported in the case of clathrin-mediated mAβ42 internalization (Hu et al., 2009). The mode of oAβ42 internalization is similar to that of ECP; however, Arf6 was not involved in the process in our study.

Imamura et al. (2011) reported that only multivalent but not monovalent HIV Tat protein could enter cells through the crosslinking of HSPGs and subsequent macropinocytosis. They showed that crosslinked HSPGs can recruit active Rac1 to lipid rafts, followed by the induction of macropinocytosis (Fan et al., 2007). The finding that monomeric Tat protein incapable of HSPG crosslinking is not internalized is compatible with our finding that mAβ42 scarcely entered cells. The similar dependence of oligomeric Aβ42 on HSPGs suggests that oAβ42 can also crosslink HSPGs and be endocytosed alongside syndecan. Letoha et al. (2019) found that syndecan-3, a neuron-specific syndecan isoform, facilitates not only cellular uptake but also fibrillization of Aβ42. In line with their findings, we observed that, at a low temperature, Aβ42 attached to the cell periphery as aggregates. The prolonged attachment to the cell surface covered with syndecan might facilitate the fibrillization of Aβ42. The fibrillated Aβ42 can no longer enter cells.

A previous study reported that astrocytes internalize both monomeric and oligomeric Aβ42, and another study showed that the monomer but not the oligomer is preferentially internalized (Omtri et al., 2012; Li et al., 2014). In our research, mAβ42 was the major species in the prepared samples, as evidenced by PAGE. Nonetheless, when samples without oAβ42 were applied, only a trace amount of Aβ42 was found in the cells, in contrast to the sample containing oAβ42, indicating that oAβ42 preferentially enters cells. The difference may stem from the various types of cells and diverse experimental conditions, since the preference will be defined by the repertories of cell surface receptors, and dozens of receptors have been reported as candidates for Aβ42 (Jarosz-Griffiths et al., 2016). Recently, Marshall et al. (2020) applied oAβ42 to primary neurons from the hippocampus of P0–P1 rats and found that oAβ42 was associated with the cell surface at the timepoint of 1 h. It was internalized *via* clathrin-dependent endocytosis at a later stage of cell culture.

Amylin is a 37-amino-acid peptide produced by pancreatic beta cells, which causes islet cell damage in its fibrillar form. An experiment using EIPA or wortmannin demonstrated that both oligomeric and monomeric amylin peptides are endocytosed by cells *via* the macropinocytosis pathway (Clark and Nilsson, 2004; Trikha and Jeremic, 2011, 2013). Notably, in the early stage, monomeric amylin is internalized by clathrin endocytosis, although oligomers enter cells regardless of the timepoint. Furthermore, in the case of amylin, it has been reported that it is detoxified when taken up by cells since the amyloid form of amylin bound to the cell surface exerts cytotoxicity.

The detailed fate of endocytosed oAβ is not yet clarified. However, massive accumulation of Aβ outside and inside the cells might implicate that the degradation system, including lysosome, cannot work properly in the AD brain. If it is the case, an impaired lysosomal function might link with neuronal cell death. Given that the internalization of oAβ is the upstream event of such lysosomal impairment, any method that can impede macropinocytosis of oAβ is of clinical relevance.

Our study has several limitations. First, our results obtained from *in vitro* assay using culture cells cannot faithfully reflect the pathological process that has taken place in a diseased brain. However, if its supposed time dependence and wide dose dependence are taken into consideration, it is conceivable that a fraction of senile plaque components enter the cells in the long-term, as shown in this study. Second, we could not specifically isolate oAβ despite the effort to purify it; instead, we used a mixture of monomeric and oligomeric Aβ as oAβ. Nonetheless, the lower relative abundance of oAβ42 in samples used as oAβ42 supports our conclusion, as mentioned above.

In conclusion, we herein report the preferential entry of oAβ42 over mAβ42 into several types of cells, including neuronal cells, in a time- and dose-dependent fashion. The mode of internalization, although not exclusive, is macropinocytosis, which depends on HSPGs and lipid rafts. The requirement for Rac1 activation suggests that the entry of oAβ42 is an active process. Further delineation of the process of oAβ42 internalization and the biological implications of intracellular Aβ42 might provide information on the development of novel types of AD therapies.

## Data Availability Statement

The original contributions presented in the study are included in the article/Supplementary Materials, further inquiries can be directed to the corresponding author.

## Author Contributions

KN and TT designed the study. MN, YY, and HMo provided advice to KN. KN, KM, and NH performed the experiments. KN and TT analyzed the data. KN wrote the first draft of the manuscript. HMa supervised the study. All authors contributed to the article and approved the submitted version.

## Conflict of Interest

The authors declare that the research was conducted in the absence of any commercial or financial relationships that could be construed as a potential conflict of interest.

## Publisher’s Note

All claims expressed in this article are solely those of the authors and do not necessarily represent those of their affiliated organizations, or those of the publisher, the editors and the reviewers. Any product that may be evaluated in this article, or claim that may be made by its manufacturer, is not guaranteed or endorsed by the publisher.

## Supplementary Materials

Supplementary Figure 1 The monomeric or oligomeric status of Aβ42 was investigated using confocal microscopy and Tricine gel. **(A)** Neuro2A cells were treated with mAβ42 and oAβ42 at concentrations of 1, 2, and 5 μM for 90 min at 37°C, and then observed by confocal microscopy. Scale bar = 10 nm. **(B)** Graph of the levels of internalization of oAβ42 or mAβ42. Data are presented as mean ± SEM. a.u. = arbitrary units. Significance was established by Student’s t-test. *****p* < 0.0001. **(C)** Next, both mAβ42 and oAβ42 were subjected to 16.5% Tris-Tricine gel to check the monomeric or oligomeric status. The gel was observed on a UV (312 nm) illuminator.

Supplementary Figure 2 Effects of inhibitors on the internalization of oAβ42 in SH-SY5Y cells. **(A)** SH-SY5Y cells were mock-treated or pretreated with 80 μM EIPA, 300 nM wortmannin, or 80 mM dynasore for 1 h, followed by 5 μM oAβ42, 2.5 mg/ml dextran, and 50 μg/ml transferrin for an additional 90 min at 37°C. Confocal microscopy in the presence of the macropinocytosis inhibitors compared with the controls. **(B)** Internalization of oAβ42 and dextran was investigated in four different cell lines: Neuro2A, SH-SY5Y, HEK293T, and HeLa cells. These cells were treated with 5 μM TMR-oAβ42 (red) and 2.5 mg/ml FITC-dextran (green) for 90 min. Nuclei were visualized with DAPI (blue). Yellow indicates colocalization. **(C)** Graph of the levels of internalization of oAβ42 by different cells. Data are presented as mean ± SEM. a.u. = arbitrary units. Scale bars = 10 μm.

## Abbreviations

AD: Alzheimer’s disease
Aβ: amyloid beta
ANOVA: analysis of variance
CTB: cholera toxin B subunit
DAPI: 4’,6-diamidino-2-phenylindole
DMEM: Dulbecco’s Modified Eagle Medium
EIPA: 5-(N-ethyl-N-isopropyl)-amiloride
HFIP: 1,1,1,3,3,3-hexafluoro-2-propanol
HSPG: heparan sulfate proteoglycan
MβCD: methyl-β-cyclodextrin
NFT: neurofibrillary tangle
oAβ: oligomeric amyloid beta.
