# Supplementary figures and images for: Amyloid Beta Is Internalized via Macropinocytosis, an HSPG- and Lipid Raft-Dependent and Rac1-Mediated Process
Source: Front Mol Neurosci. 2022 Feb 11;15:804702. doi: 10.3389/fnmol.2022.804702 (PMC9524458; doi:10.3389/fnmol.2022.804702)

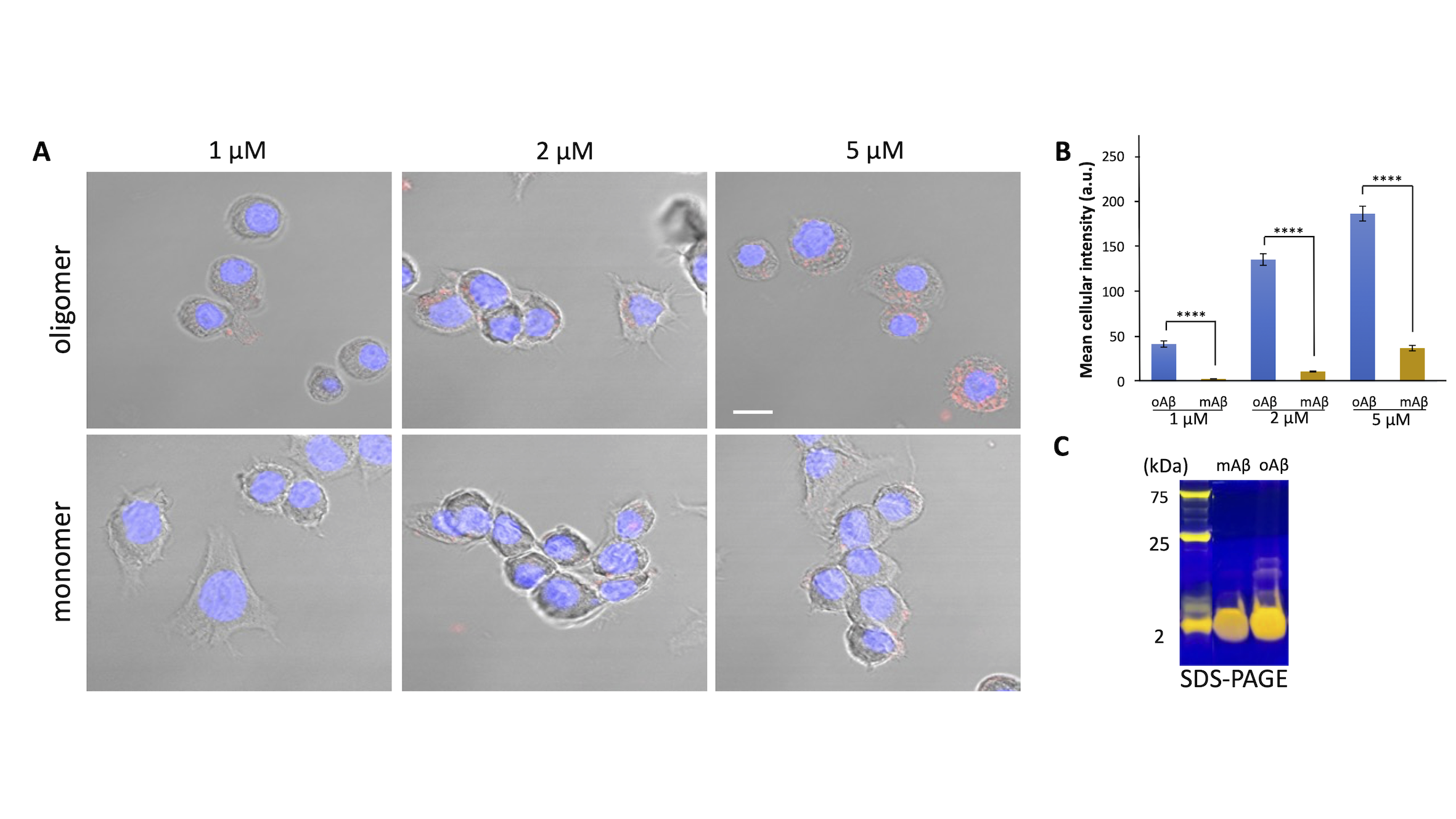

Supplement: Supplementary Figure 1 — The monomeric or oligomeric status of Aβ42 was investigated using confocal microscopy and Tricine gel. (A) Neuro2A cells were treated with mAβ42 and oAβ42 at concentrations of 1, 2, and 5 μM for 90 min at 37°C, and then observed by confocal microscopy. Scale bar = 10 nm. (B) Graph of the levels of internalization of oAβ42 or mAβ42. Data are presented as mean ± SEM. a.u. = arbitrary units. Significance was established by Student’s t-test. ****p < 0.0001. (C) Next, both mAβ42 and oAβ42 were subjected to 16.5% Tris-Tricine gel to check the monomeric or oligomeric status. The gel was observed on a UV (312 nm) illuminator. [file Image_1.TIFF]

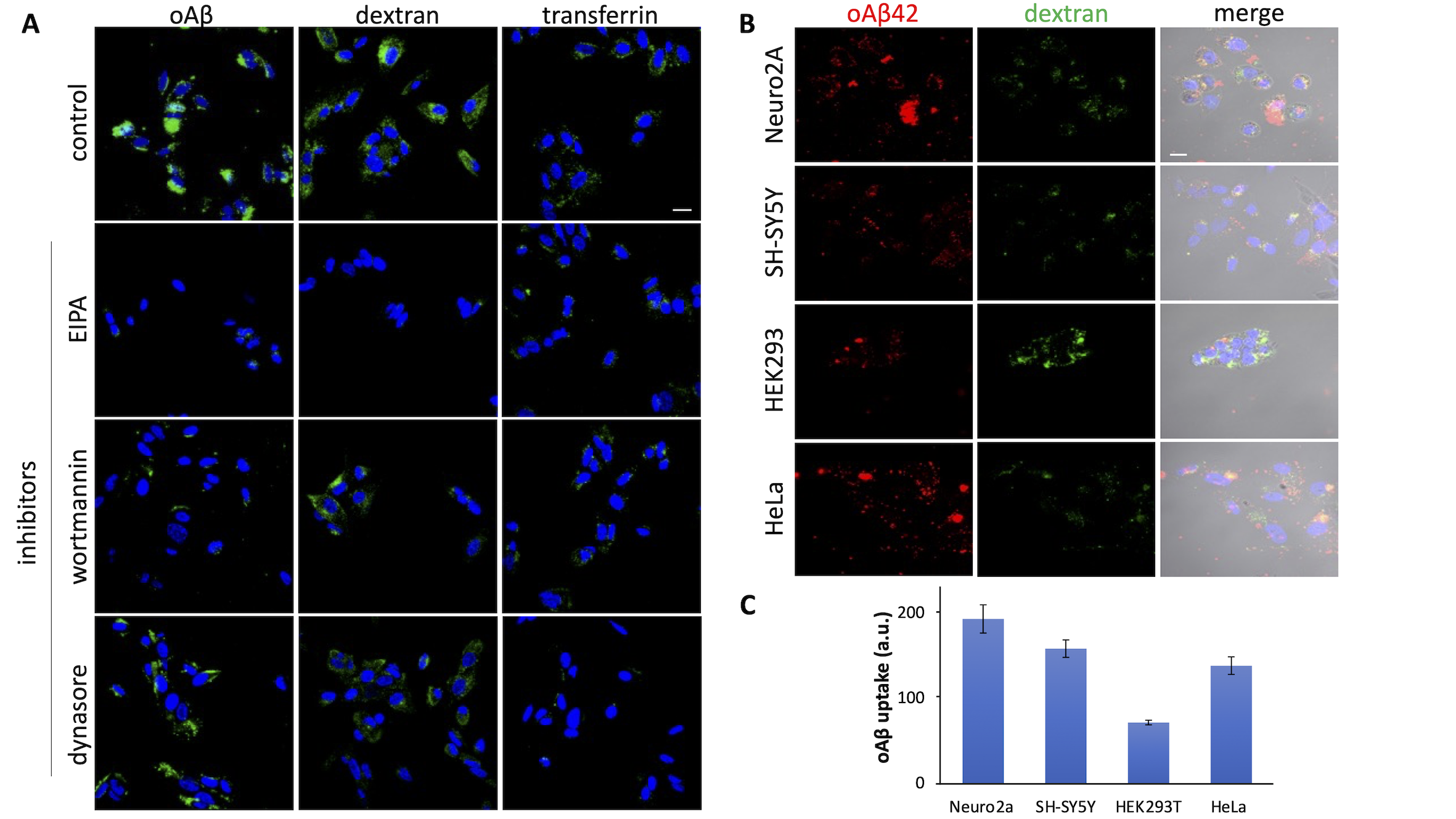

Supplement: Supplementary Figure 2 — Effects of inhibitors on the internalization of oAβ42 in SH-SY5Y cells. (A) SH-SY5Y cells were mock-treated or pretreated with 80 μM EIPA, 300 nM wortmannin, or 80 mM dynasore for 1 h, followed by 5 μM oAβ42, 2.5 mg/ml dextran, and 50 μg/ml transferrin for an additional 90 min at 37°C. Confocal microscopy in the presence of the macropinocytosis inhibitors compared with the controls. (B) Internalization of oAβ42 and dextran was investigated in four different cell lines: Neuro2A, SH-SY5Y, HEK293T, and HeLa cells. These cells were treated with 5 μM TMR-oAβ42 (red) and 2.5 mg/ml FITC-dextran (green) for 90 min. Nuclei were visualized with DAPI (blue). Yellow indicates colocalization. (C) Graph of the levels of internalization of oAβ42 by different cells. Data are presented as mean ± SEM. a.u. = arbitrary units. Scale bars = 10 μm. [file Image_2.TIFF]
